# Experiences and parenting practices of child caregivers in light of the Nurturing Care framework

**DOI:** 10.1590/0034-7167-2024-0473

**Published:** 2026-10-26

**Authors:** Thayná Rhayanne Andrade Rufino da Silva Costa, Ana Paula Esmeraldo Lima, Daniela Tavares Gontijo, Weslla Karla Albuquerque de Paula, Maria Wanderleya de Lavor Coriolano-Marinus

**Affiliations:** I Universidade Federal de Pernambuco. Recife, Pernambuco, Brazil

**Keywords:** Desarrollo Infantil, Responsabilidad Parental, Aprendizaje, Cuidado del Niño, Educación en Salud., Child Development, Parenting, Learning, Child Care, Health Education

## Abstract

**Objectives:**

to describe the experiences and parenting practices of primary caregivers linked to the Family Health Strategy regarding the development of children aged 0 to 6 years based on the Nurturing Care Framework.

**Methods:**

a qualitative study was conducted with 13 caregivers of children followed by the Family Health Strategy in the municipality of Recife, Pernambuco. Data collection took place from December 2022 to April 2023, through semi-structured interviews. Data were subjected to thematic analysis.

**Results:**

five thematic categories were systematized, arranged according to the Nurturing Care Framework: “Good health”; “Adequate nutrition”; “Responsive caregiving”; “Early learning”; and “Safety and security”.

**Final Considerations:**

parental experiences and practices were considered in all areas of the framework, even within the context of vulnerability experienced by families. Positive parenting practices require a reorganization of healthcare services, starting with the implementation of interventions that encourage holistic child development.

## RESUMEN

**Objetivos:**

describir las experiencias y prácticas parentales de los cuidadores primarios vinculados a la Estrategia de Salud Familiar en relación con el desarrollo infantil de niños de 0 a 6 años con base en el *framework Nurturing Care*.

**Métodos:**

estudio cualitativo con 13 cuidadores de niños que participan en la Estrategia de Salud Familiar en el municipio de Recife, Pernambuco. La recolección de datos se realizó entre diciembre de 2022 y abril de 2023 mediante entrevistas semiestructuradas. Los datos se sometieron a un análisis temático.

**Resultados:**

se sistematizaron cinco categorías temáticas, ordenadas según el *framework Nurturing Care*: “Buena salud”; “Nutrición satisfactoria”; “Cuidado responsivo”; “Aprendizaje temprano”; y “Protección y seguridad”.

**Consideraciones finales:**

las experiencias y prácticas parentales se consideraron en todas las áreas del marco, incluso en el contexto de vulnerabilidad de las familias. Las prácticas parentales positivas requieren una reorganización de los servicios de salud, comenzando con la implementación de intervenciones que fomenten el desarrollo integral del niño.

## INTRODUCTION

Parental support from different sectors of society is relevant for responsive interaction between parents and children^(1)^. Global strategies, such as the Nurturing Care Framework, launched by the World Health Organization in 2016, consider the integration of different components to support parenting skills and improve overall child development, based on the Sustainable Development Goals^(2)^. Its structure is composed of five priority and integrated axes: Good health; Adequate nutrition; Responsive caregiving; Safety and security; and Opportunities for early learning^(1,2)^.

According to the Nurturing Care Framework, good health encompasses children’s physical and emotional well-being, achieved through parental vigilance and responsiveness to their daily needs in an affectionate and appropriate manner. This axis also includes maternal well-being, such as immunization, family planning, mental health support, and prenatal and postpartum care^(2)^. Adequate nutrition involves promoting adequate nutrition through support for breastfeeding, healthy eating, and nutrient supplementation from the gestational period through birth and throughout a child’s life. Inadequate nutrition and micronutrient deficiencies can affect child development^(2)^. Responsive caregiving encompasses the relationship between caregivers and children, and can be expressed through feelings, eye contact, sounds, gestures, and physical actions of affection^(2)^. Learning is an innate mechanism of human beings, and in the early years, the acquisition of these skills depends on interaction with people, through teaching, gestures, demonstrations, imitations, and play^(2)^. Safety and security involve guaranteeing protection for children through policies that support income, promote healthy family environments and leisure activities in urban and rural areas, and prevent family violence, including physical violence, punishment, threats, or abandonment^(2)^.

In the Brazilian context, child health promotion monitoring is carried out by Primary Care, with multidisciplinary actions from the Family Health Strategy (FHS)^(3,4)^. Although actions to promote child health have been systematized in Primary Care since the creation of the Family Health Program, the implementation of integrated child care actions, as proposed by Nurturing Care, is not yet part of the FHS’s work due to difficulties such as work overload, staff shortages, and high demand for services, which were highlighted by nurses as some of the factors hindering positive parenting promotion^(5)^.

Positive parenting practices are based on stability, love, and respectful relationships between caregivers and children, while negative parenting practices are based on inconsistency, physical violence, and punishment methods as a form of parenting^(6)^. Negative parenting practices have been linked to increased levels of childhood behavioral problems. In many situations, the use of violent disciplinary practices may be the result of parental anger and frustration, or a lack of knowledge of non-violent parenting practices as parenting strategies^(7)^. Parenting practices can be modified through support in developing parenting skills from health and education professionals. Understanding the beliefs and cultural factors in which families are involved is crucial for integrated interventions that support child development. Therefore, knowledge about parental experiences and practices in Brazilian sociocultural contexts becomes relevant, starting from an understanding of parental relationships as influencing child care and protection, based on existing and consolidated policies such as the FHS so that effective parental interventions can be tailored to caregivers’ needs and be effective.

The experiences and parenting practices of caregivers of children aged 0 to 6 years were chosen because they encompass early childhood (the first six years of life). This age range has been prioritized in public policies focused on comprehensive care, particularly in the areas of health, education, and social assistance^(1-4)^.

The study was guided by the following research question: what are the experiences and parental practices of primary caregivers regarding the child development of children aged 0 to 6 years based on the Nurturing Care Framework?

## OBJECTIVES

To describe the experiences and parenting practices of primary caregivers linked to the FHS regarding the child development of children aged 0 to 6 years based on the Nurturing Care Framework.

## METHODS

### Ethical aspects

The research complied with Resolutions 466/2012 and 510/2016 of the Brazilian National Health Council. The study was approved by the *Universidade Federal de Pernambuco* Research Ethics Committee.

### Theoretical-methodological framework

The study was anchored in the Nurturing Care Framework^(2)^, based on its five axes: Good health; Adequate nutrition; Responsive caregiving; Opportunities for early learning; and Safety and security.

The study was guided by the COnsolidated criteria for REporting Qualitative research to ensure adherence to methodological rigor criteria^(8)^.

### Study design

This is a descriptive, exploratory, qualitative study^(9)^, carried out using the Nurturing Care Framework-a bioecological perspective for parental support interventions aimed at comprehensive child development^(2,10)^.

### Study location

The research was conducted at a Family Health Unit (FHU) in the municipality of Recife, in northeastern Brazil. The unit has three family health teams and two oral health teams, along with professionals from the family health support center, who provide care in Primary Care and in Primary Care emergencies^(10)^.

### Study participants

The sample consisted of 13 primary caregivers linked to the FHU who had children aged 0 to 6 years. Primary caregivers of children with special needs were excluded due to already evident developmental and learning impairments. The sample was intentionally selected, by convenience, from among caregivers who attended the unit for well-child visits, vaccinations, or other appointments, as well as through referrals from community health workers during home visits. If they could not conduct the interview on the same day at the health unit, it would be scheduled at a non-governmental organization in the same community, on a day and time convenient for participants. Participants were briefed on all stages of the study, and their agreement was confirmed by signing the Informed Consent Form.

### Data collection

Data were obtained through individual interviews conducted between December 2022 and April 2023. Nine visits were made to the FHU for data collection, in addition to three preliminary visits to familiarize oneself with the health team, the population, and the context of the study. Semi-structured interviews were conducted by the principal investigator (master’s student) and a research assistant (undergraduate student), who had been previously trained in the study’s techniques and the principles of qualitative research. The interviews were conducted individually and recorded without the presence of non-participants, and lasted approximately 20 to 30 minutes. The technique used was the semi-structured interview, with the aid of a script with open-ended questions and a field diary for recording the researcher’s impressions immediately after the end of each interview.

### Data analysis

The interviews were transcribed, returned to participants via WhatsApp^®^, and analyzed using thematic analysis, following these stages: transcription; initial data coding; subtopic formation; topic formation; and categorization^(11)^.

Initial coding was performed in a Google Docs^®^ file for line-by-line coding. The initial codes were discussed by the principal investigator, research assistant, and advisor for systematization, organization, and creation of subtopics and topics in periodic meetings. In the categorization stage, the data were organized from a deductive perspective, within the Nurturing Care Framework components.

## RESULTS

### Participant characterization

Thirteen primary caregivers of children aged 0 to 6 years participated in the study. All were female, with 12 being mothers and one grandmother. Their ages ranged from 18 to 46 years. Regarding education and income, most had completed high school (ten participants) and had a monthly income of one to two minimum wages. Of the total interviewed, eight declared themselves single, and five married. Concerning religion, five declared themselves Catholic; two declared themselves Christian/Evangelical; and six declared themselves to have no religion. In relation to housing, most reported living with four people. As for income, most live on an average of one and a half minimum wages. Two families reported that their family income was less than one minimum wage. In terms of race/color, 11 participants identified as Black or mixed-race. Regarding childcare time, seven caregivers reported not working and caring exclusively for their child, while six caregivers reported working outside the home and caring for their children during their off-hours. Information about family support was mentioned only by the caregivers who worked. The caregivers who worked had a support network characterized by grandparents, aunts, parents, and godmothers of children.

When analyzing participant sociodemographic data, it was identified that women were the primary caregivers living in a context of precarious housing and sanitation conditions, limited income, and constant exposure to risks.

Children’s ages varied, with four being under 6 months old; four being 1-2 years old; and eight being 2-5 years old. Three caregivers had two children under 6 years old.

### Qualitative data characterization

The five categories presented are based on the pillars of the Nurturing Care Framework (Good health; Adequate nutrition; Responsive caregiving; Opportunities for early learning; and Safety and security), as described in Chart 1. The pillars of the Nurturing Care Framework were explored from the topics that emerged from individual interviews, which explored the experiences and parenting practices of caregivers that influence the development of children aged 0 to 6 years assigned to the FHS.

**Chart 1:** Categories with topics and subtopics based on the experiences and practices of parents and mothers of children aged 0 to 6 years, Recife, Pernambuco, Brazil, 2023

Nurturing Care Framework axis	Topics	Subtopics	Statements
**Good health**	1. Health situation	- Pregnancy planning - Fear of motherhood and experiences	*“This pregnancy was a shock to me; it was a real shock. It came with a lot of stress”.* (I4) (1-month-old child) *“It was very difficult because I never wanted to be a mother. I always thought it was beautiful, but the truth is I never wanted the responsibility of having a child, and now I have to experience all of this”.* (I5) (Children aged 1 and 3 years)
**Good health**	2. Coping with the pandemic	- Changes in people’s and children’s routines - Changes in the interpersonal and social relationships of caregivers and children - Changes in children’s school life - Protective and preventative measures against the virus - Fear of contamination during the pandemic	*“He came during the pandemic, because I had one, lost one, had M, and in this pregnancy, I thought I had COVID. In the beginning, I had shortness of breath, vomiting, and I didn’t think it was pregnancy, because, for me, it’s very difficult to have a pregnancy”.* (I1) (2-year-old child) *“It brought more stress because he was stressed out, wanting to go out all the time”.* (I11) (3-year-old child) *“He would teach (his schoolwork) once in a blue moon. We would teach him and nag him”.* (I6) (5-year-old child)
**Good health**	3. Child accompaniment at the healthcare service	- Access to and monitoring of healthcare services and use of a Child’s Health Record	*“I think the part about the health center could improve, especially regarding children, because it’s very difficult to get an appointment”.* (I5) (Children aged 1 and 3 years)
**Good health**	4. Child development and behavior	- Concept of child development - Parental role in promoting child development	*“I think healthy development is when she’s happy, when she has her time to play, and her time to learn, because whether we like it or not, we have to educate* [...]”. (I13) (4-year-old child) *“She already has a little book from a friend of mine that came back from a trip, but she doesn’t actually read it; she keeps trying to tear it up”.* (I2) (1-year-old child)
**Adequate nutrition**	1. Child nutrition	- Healthy nutrition for children - Breastfeeding practices	*“In the morning, when she wakes up, she has porridge that my mother makes. In the afternoon, she eats fruit, has lunch, drinks juice, has a small snack, and then at night it’s porridge again”.* (I2) (1 year old child) *“He breastfeeds and takes formula at night. Because he doesn’t stay on breast milk alone”.* (I8) (1-month-old child)
**Responsive caregiving**	1. Caregiver/child relationship	-Caregiver-child relationship and child encouragement	*“They really need us”.* (I1) (2-year-old child) *“If he’s putting the puzzle together, I go over and see that he’s finished. I always say, ‘Very good, congratulations!’”.* (I13) (4-year-old child)
**Responsive caregiving**	2. Family interaction and care for the child and support network	- Understanding parenting practices in daily care - Support network	*“I read him a story, then he asks for two, and I say, ‘No, it’s one story, okay?’, then I put on a lullaby, and then he falls asleep”.* (I11) (3-year-old child) *“He plays with his father. When everyone is sleepy, we go to sleep”.* (I6) (5-year-old child) *“Either I play with him or he goes to his godmother’s house”.* (I11) (3-year-old child) *“I don’t have my father’s support! He should take responsibility for raising children and caring for them, at least on weekends”.* (I5) (Children aged 1 and 3 years)
**Responsive caregiving**	3. Feelings about parenthood	- Feelings in childcare	*“I think that to be a mother, you have to have patience, you have to have a little experience”.* (I4) (1-month-old child) *“Enjoyable, but very hard work. Total dedication”.* (I1) (2-year-old child)
**Opportunities for early learning**	1. Learning relationship between caregivers and children in the home setting	- Caregiver involvement in promoting learning - Children’s activities in promoting learning opportunities and stimulating development	*“I think we have to teach. We have to have the patience to teach* [...] *otherwise he won’t learn on his own”.* (I7) (4-month-old child) *“Often, learning happens through play, so while not all games, some are indeed very stimulating”.* (I12) (4-year-old child)
**Opportunities for early learning**	2. Caregivers’ relationship with the school context	- The role of the school in promoting children’s development and learning - Participation and support of caregivers in children’s school life and learning experiences and practices - Accessibility to educational institutions	*“She’s very advanced when it comes to eating. She’s very didactic when she needs to eat. She gets food herself, she talks, she says what she wants. If she’s hungry* [...] *these things are much more pronounced when it comes to a child who goes to daycare and one who doesn’t, which is their case”.* (I5) (Children aged 1 and 3 years) *“Whenever there are meetings, she’s always present, always engaging with the teachers, because there’s a group of teachers who always report what happened and what didn’t happen. She’s always looking for new things”.* (I12) (4-year-old child)
**Safety and security**	1. Financial resources and leisure	- Practices of activities and leisure spaces between family and child	*“No, sporadically. I have free time and money, we’ll go”.* (I5) (Children aged 1 and 3 years) *“The problem is time. I only have time on weekends. When I do have time on weekends, what do I usually try to do? Take care of the house, clean, cook, all that stuff, which is quite difficult”.* (I5) (Children aged 1 and 3 years)

### Category 1 - Good health: understanding health practices with children

In the “Good Health” category, the data highlighted maternal health conditions from pregnancy planning and acceptance to caregiving relationships, such as hygiene and sleep routines. The context of the COVID-19 pandemic was marked by fear of contagion, and coping measures focused on prevention and influencing children’s routines, such as greater restrictions on the home environment and a preference for outdoor activities. Furthermore, there were changes in children’s social, behavioral, and school life. The need for improved access to healthcare services was identified, including well-child visits and the use of a Child’s Health Record. Mothers recognized the importance of the record for documenting children’s data and for its informative content, although they described difficulties in reading the information provided.

### Category 2 - Adequate nutrition: understanding practices of proper nutrition for children

In the “Adequate nutrition” category, caregivers reported on their daily practices regarding infant feeding and breastfeeding. Among the participating children, five were in the 0-6 month age range, but only three caregivers reported on breastfeeding practices. Among these, one even reported that exclusive breastfeeding was not sufficient. The use of processed foods and sugars as detrimental factors to a healthy diet was present in the reports. Only one caregiver mentioned reducing the consumption of foods high in sugar and processed foods.

### Category 3 - Responsive caregiving: understanding caregivers’ knowledge and practices in childcare

In the category of “Responsive caregiving”, caregivers mentioned perceiving children’s needs based on understanding their dependence in the attachment and sensitivity relationship, as well as mentioning positive parenting practices, such as encouraging them to face cognitive challenges like assembling a puzzle, and reading and storytelling. The support network was mentioned in relation to participation in activities of daily living, being composed of family members, relatives, and neighbors. However, some interviewees mentioned the absence of members, such as the absence of a “father” in daily support.

### Category 4 - Opportunities for early learning: understanding caregiver practices that enable learning opportunities

In the “Opportunities for early learning” category, caregivers perceived that their interactions in play and daily activities contributed to children’s learning. Furthermore, they mentioned the school’s participation and importance in this process, highlighting family engagement in school activities.

### Category 5 - Safety and security: understanding children’s income, leisure, and safety conditions

In the “Safety and security” category, caregivers mentioned the influence of financial resources on families’ leisure time. Another aspect is the families’ financial situation, with financial resources particularly focused on subsistence.

## DISCUSSION

The results present the experiences and parenting practices of caregivers of children aged 0 to 6 years registered with the FHS. The main experiences narrated were based on daily interactions of care with children, including the parental role superimposed on other demands of daily life, such as housework, dealing with unemployment, or working long hours. Parenting practices were based on accounts of actions involving conversations, play, and storytelling. The context is directly related to health outcomes. It is important to emphasize that unfavorable environmental and income conditions for children and families may be related to negative outcomes in child development.

Caregivers, most of whom were children’s mothers, mentioned feelings of satisfaction with motherhood, such as love, happiness, strength, patience, responsibility, and pleasure in caring for children. However, feelings of abdication and exhaustion were also evident. This aspect was related both to mothers who worked outside the home and to the accumulation of domestic activities and lack of shared domestic responsibilities with their partners.

This aspect is corroborated by studies that point to higher rates of stress, tiredness, and fatigue in mothers who have both professional and family responsibilities. Women are more prone to resorting to authoritarian parenting practices and punishments. All of this can lead to feelings of guilt and inability to perform multiple roles effectively^(12)^.

The results of this study were arranged into categories that are consistent with the strategic axes of the Nurturing Care Framework already mentioned in this study. Together, these provide comprehensive care for children, thus protecting children from the risks that threaten intellectual disability and the impacts on intergenerational cycles^(2)^.

Regarding the health category, reports highlighted interactions between caregivers and children, and the monitoring of child development in healthcare services, from prenatal care onwards, which was surrounded by feelings of uncertainty and fear during the COVID-19 pandemic.

Monitoring child development through well-child visits was a reality for participants, although difficulties in access and limitations in using a Child’s Health Record as a tool for parental literacy were mentioned. Studies indicate that the pandemic primarily affected children living in vulnerable situations, as it hindered access to healthcare and education services and reduced family income. In education, schools were closed due to the measures taken to combat the pandemic, negatively impacting children’s nutritional status and learning. Often, the school is the one that provides daily meals and opportunities for learning and interaction among peers^(12)^.

Furthermore, studies show that there has been a disconnect between family and school, especially when education is conducted remotely. Many of these children did not have internet access. During this period, poverty also increased and employment decreased, leading to overcrowding in the home and increased stress, which can hinder caregivers from providing responsive and consistent care^(13,14)^.

Another aspect explored was the caregivers’ understanding of child development and the factors that can hinder a child’s development. From this perspective, the concept of development as a healthy, happy child with good eating habits (breastfeeding, adequate food consumption) was highlighted, as well as responsive practices on the part of caregivers, such as affection and routines. In relation to children’s behavior, the caregivers mentioned perceptions about motor skills, such as being very active, as well as characteristics related to cognitive development, such as paying attention and enjoying books and school, in addition to behavioral characteristics, such as obedience to caregivers.

Regarding the “Adequate nutrition” category, the caregivers in this study mentioned the main nutrition-related practices used daily, such as breastfeeding and complementary feeding. Infant nutrition is a crucial aspect for comprehensive child development. Studies indicate that inadequate nutrition can affect various aspects of a child’s life, from health issues to risks to overall child development, leading to behavioral problems^(15)^. Nutritional deficiencies can result in poor school performance, cognitive deficits, and chronic diseases in adulthood, which can persist into subsequent generations, potentially causing long-term impacts on the health and socioeconomic conditions of these children^(16)^.

From the perspective of responsive caregiving, in the studied reality, responsive actions were found in bedtime storytelling and book reading, in which children interacted and asked for more stories or even created their own stories, in addition to the habit of conversing during daily interactions, using age-appropriate games such as mime, color games, dance games, and songs.

These parenting practices directly influence child development, especially in the cognitive and learning aspects of children, which corresponds to responsive caregiving practices^(1,6,17)^. Responsive caregiving stems from the involvement and assistance of parents or other caregivers in their relationships and interactions with children, obtained through daily interactions in a sensitive and attentive manner. This creates opportunities for interaction and learning with the environment, objects, and people, and is fundamental for achieving healthy, holistic child development^(18)^.

From the perspective of childcare and parenting practices, perceptions of how caregivers can influence or assist in child development are highlighted. However, access to information does not translate into its broad understanding and applicability in parental care, for reasons that include overload and stress, limited sources of support (including the role of men and other family members), social determinants (such as income and education), and a lack of motivation for parental learning^(19)^.

Implementing interventions that support caregivers in positive parenting practices and the creation of a safe and healthy environment are essential in healthcare professionals’ professional practice, as they can bring numerous benefits to the health of children and their families, especially regarding the reduction of negative events and outcomes^(20)^. However, in the Brazilian context, there are difficulties in implementing guidelines and interventions related to acquiring positive practices for child development within the context of Primary Care. Professionals encounter barriers to addressing sensitive topics that require practical skills, such as respectful parenting practices, making continuous improvement and education necessary to enhance actions focused on childcare^(5,21)^.

Among the parenting practices reported by caregivers, positive parenting practices stood out, such as the use of play with drawings and educational games, music, and storytelling in children’s daily routine. A relevant fact is that, despite not being aware of practices that encourage reading and learning, it was observed that some caregivers do encourage reading through contact with books. There were no reports of other types of negative practices, such as physical punishment and violence.

Studies indicate the benefits of interventions aimed at stimulating parenting practices in order to promote knowledge and skills in caregivers, improving parental knowledge and interactions between parents and children. The results also impact children’s developmental scores, with improved cognitive, motor, and language skills^(17,22)^.

Healthcare services and early childhood education have been influential in caregivers’ experiences and parenting practices, with monitoring and oversight of health and learning. Studies show that parenting practices are essential for creating an environment conducive to child development, as they act as factors of opportunity and stimulation^(1,17,18)^.

In the “Opportunities for early learning” category, caregivers perceived their role in children’s learning, portraying caregiving actions as teachings and playful activities for children’s learning. Another aspect was the perception of the importance of maintaining school support, although the lack of time for closer and more engaged monitoring was mentioned by caregivers who were employed.

Parental practices such as storytelling and engaging with books can facilitate learning and foster connection between caregiver and child, which can provide motivation and spark increasing interest in children. Studies indicate that among the benefits of play activities are the acquisition of cognitive, linguistic, and motor skills, which are directly related to emergent learning, social competency promotion, and behavior and emotion self-regulation promotion^(23)^.

From the perspective of school support, it is possible to identify that school is an extension of the family environment as a means of learning. Therefore, family engagement and support in learning activities are crucial for children’s learning and development^(24)^.

In the “Safety and security” category, even with the vulnerability and economic constraints, some caregivers seek to promote leisure time in free environments found in their own community and surrounding areas. Parental interventions, although diverse in middleand low-income countries, show promising results, particularly from behavior change strategies using diaries, screen reminders, telephone contacts, encouraging segments, and the use of interactive drama (theater and interactive techniques)^(25)^.

In our study, positive parenting practices and responsive caregiving were found through accounts related to knowledge built through their experiences of motherhood and in their relationships with family members. These actions need to be guided and expanded in the daily lives of these families, with the support of healthcare and education professionals in an integrated way. Therefore, the training of healthcare professionals to implement interventions that promote responsive caregiving can generate gains not only for individuals, but for the entire community, following a bioecological perspective advocated by the Nurturing Care Framework, used as a theoretical reference for this study.

### Study limitations

Among the study’s limitations, the data collection period stands out, given that inviting and recruiting participants faced difficulties due to the fact that well-child visits at the FHU were intended for children aged 0 to 2 years. Other recruitment strategies, such as inviting caregivers who sought care at the unit for other reasons, such as vaccinations and emergencies in the case of older children, may have resulted in additional demands related to parenthood during critical caregiving moments. The exclusion of caregivers with children with special health needs was justified by the additional burden and greater demands of parenthood on caregivers facing these challenges. We acknowledged that this is a limitation of the study and could be a field of investigation in studies on parental experiences.

### Contributions to nursing, health, or public policy

This work contributes to the discussion of positive parenting practices and to the expansion of parenting support interventions based on the experiences and prior knowledge of caregivers. Parenting support actions should be a priority for promoting child development, through the integration of components that go beyond biological models and adopt participatory and intersectoral approaches capable of breaking intergenerational cycles.

## FINAL CONSIDERATIONS

The experiences and parenting practices of primary caregivers of children aged 0 to 6 years served by the FHS aligned with the axes of the Nurturing Care Framework, even in the absence of its incorporation by the FHS in the Brazilian context, due to the vulnerable situation experienced by these families. It can be inferred that positive parenting practices supporting parenting require a reorganization of healthcare services through the implementation of interventions that encourage responsive caregiving and holistic child development.

Parenting was described through positive practices, highlighting the use of play, music, and storytelling in children’s daily routines. Responsive caregiving was described through the perception and understanding of how to respond to children’s actions in a practical and timely manner, through sensitive and responsive interactions. Caregivers mentioned the role of healthcare services most strongly related to the of “good health” and “adequate nutrition” axes, such as health actions like vaccination and medical care. The school was perceived as a supportive environment for development and learning situations.

## Data Availability

The research data are available only upon request.
