# Supplementary material for: Experiences and parenting practices of child caregivers in light of the Nurturing Care framework
Source: Rev Bras Enferm. 2026 Oct 26;79:e20240473. doi: 10.1590/0034-7167-2024-0473 (PMC13719130; doi:10.1590/0034-7167-2024-0473)
Supplement: Translation

# Translation: Vivências e práticas parentais de cuidadoras de crianças à luz da *framework Nurturing Care*

**DOI:** 10.1590/0034-7167-2024-0473pt

**Authors:** Thayná Rhayanne Andrade Rufino da Silva Costa, Ana Paula Esmeraldo Lima, Daniela Tavares Gontijo, Weslla Karla Albuquerque de Paula, Maria Wanderleya de Lavor Coriolano-Marinus

**Affiliations:** I Universidade Federal de Pernambuco. Recife, Pernambuco, Brasil

**Keywords:** Desenvolvimento Infantil, Poder Familiar, Aprendizagem, Cuidados da Criança, Educação em Saúde.

## Abstract

**Objetivos:**

descrever as vivências e práticas parentais de cuidadores primários vinculados à Estratégia Saúde da Família sobre o desenvolvimento infantil de crianças de 0 a 6 anos a partir da *framework Nurturing Care.*

**Métodos:**

estudo qualitativo, com 13 cuidadoras de crianças acompanhadas pela Estratégia Saúde da Família do município do Recife, Pernambuco. A coleta de dados aconteceu de dezembro de 2022 a abril de 2023, mediante entrevistas semiestruturadas. Os dados foram submetidos à análise temática.

**Resultados:**

foram sistematizadas cinco categorias temáticas, dispostas a partir da *framework Nurturing Care:* “Boa saúde”; “Nutrição satisfatória”; “Cuidados responsivos”; “Aprendizagem precoce”; e “Proteção e segurança”.

**Considerações Finais:**

as vivências e práticas parentais foram contempladas em todos os eixos da *framework*, mesmo mediante o contexto de vulnerabilidade vivido pelas famílias. As práticas parentais positivas requerem uma reorganização dos serviços de saúde, a partir da implementação de intervenções que incentivem o desenvolvimento infantil integral.

## INTRODUÇÃO

O apoio parental por diferentes setores da sociedade é relevante para a interação responsiva entre pais e filhos^(1)^. Estratégias globais, como a *framework Nurturing Care*, lançada pela Organização Mundial da Saúde em 2016, consideram a integração de diferentes componentes para apoiar as competências parentais e melhorar o desenvolvimento infantil global, a partir dos Objetivos de Desenvolvimento Sustentável^(2)^. A sua estrutura é composta por cinco eixos prioritários e integrados: Saúde adequada; Nutrição satisfatória; Cuidados responsivos; Proteção e segurança; e Oportunidades de aprendizagem^(1,2)^.

De acordo com a *framework Nurturing Care*, a boa saúde envolve as condições físicas e emocionais da criança, por meio da vigilância e da resposta parental às suas necessidades diárias de forma afetuosa e apropriada. Este eixo também contempla as condições maternas, como imunização, planejamento familiar, apoio à saúde mental e atenção do pré-natal ao pós-parto^(2)^. A nutrição satisfatória envolve a promoção de uma nutrição adequada por meio do apoio à amamentação, alimentação saudável e suplementação de nutrientes desde o período gestacional até o nascimento e decorrer da vida da criança. A nutrição inadequada e a carência de micronutrientes podem afetar o desenvolvimento infantil^(2)^. Os cuidados responsivos abrangem o relacionamento entre cuidadores e crianças, e podem ser expressos por meio de sentimentos, contato visual, sons, gestos e ações físicas de afeto^(2)^. A aprendizagem é um mecanismo inato do ser humano, e nos primeiros anos, a aquisição dessas habilidades depende da interação com as pessoas, por meio de ensinamentos, gestos, demonstrações, imitações e brincadeiras^(2)^. A proteção e segurança envolvem garantia de proteção para as crianças, por meio de políticas de apoio à renda, promoção de ambientes familiares saudáveis e de lazer em áreas urbanas e rurais, e prevenção à violência familiar, incluindo violência física, castigos, ameaças ou abandono^(2)^.

Na realidade brasileira, o acompanhamento de promoção da saúde da criança ocorre pela Atenção Primária, com ações multiprofissionais da Estratégia Saúde da Família (ESF)^(3,4)^. Embora as ações de promoção da saúde da criança sejam sistematizadas na Atenção Primária desde a criação do Programa Saúde da Família, a execução de ações integradas de cuidado infantil, como proposto pela *Nurturing Care,* ainda não faz parte da atuação da ESF devido às dificuldades, como sobrecarga de trabalho, escassez de profissionais e alta demanda por atendimentos, os quais foram destacadas pelos enfermeiros como alguns dos fatores que dificultam a promoção da parentalidade positiva^(5)^.

As práticas parentais positivas são baseadas em estabilidade, amorosidade e relações de respeito entre cuidadores e crianças, enquanto as práticas parentais negativas são baseadas na inconsistência, violência física e métodos de punição como forma de educação parental^(6)^. Práticas parentais negativas têm sido relacionadas a níveis aumentados de problemas de comportamento infantil. Em muitas situações, o uso de práticas disciplinares violentas pode ser o resultado da raiva e frustração dos pais, ou falta de conhecimento de práticas parentais não violentas como estratégias de educação parental^(7)^. As práticas parentais podem ser modificadas a partir do apoio quanto às competências parentais por profissionais de saúde e de educação. O conhecimento das crenças e fatores culturais nos quais as famílias estão envolvidas é crucial para intervenções integradas que apoiem o desenvolvimento infantil. Por isso, torna-se relevante o conhecimento sobre vivências e práticas parentais em contextos socioculturais brasileiros, a partir da compreensão das relações parentais como influenciadoras do cuidado e proteção à criança, a partir de políticas já existentes e consolidadas, como a ESF, para que intervenções parentais efetivas possam ser adequadas às necessidades dos cuidadores e efetivas.

As vivências e práticas parentais de cuidadores de crianças na faixa etária de 0 a 6 anos foram escolhidas por contemplar a primeira infância (primeiros seis anos de vida). Esta faixa etária tem sido priorizada em políticas públicas voltadas aos cuidados integrais, particularmente nas áreas da saúde, educação e assistência social^(1-4)^.

O estudo foi norteado pela seguinte questão de pesquisa: quais as vivências e práticas parentais de cuidadores primários sobre o desenvolvimento infantil de crianças de 0 a 6 anos a partir da *framework Nurturing Care*?

## OBJETIVOS

Descrever as vivências e práticas parentais de cuidadores primários vinculados à ESF sobre o desenvolvimento infantil de crianças de 0 a 6 anos a partir da *framework Nurturing Care.*

## MÉTODOS

### Aspectos éticos

A pesquisa respeitou as Resoluções n^o^ 466/2012 e n^o^ 510/2016 do Conselho Nacional de Saúde. O estudo foi aprovado pelo Comitê de Ética em Pesquisa da Universidade Federal de Pernambuco.

### Referencial teórico-metodológico

O estudo foi ancorado na *framework Nurturing Care* ^(2)^, a partir dos seus cinco eixos: Boa saúde; Nutrição satisfatória; Cuidados responsivos; Aprendizagem precoce; e Segurança e proteção.

O estudo guiou-se pelo *Consolidated criteria for Reporting Qualitative Research* (COREQ) para observância de critérios de rigor metodológico^(8)^.

### Tipo de estudo

Estudo descritivo, exploratório, qualitativo^(9)^, realizado a partir da *framework Nurturing Care-*uma perspectiva bioecológica para intervenções de apoio parental com vistas ao desenvolvimento infantil integral^(2,10)^.

### Local do estudo

A pesquisa foi desenvolvida em uma Unidade de Saúde da Família (USF), no município do Recife, no nordeste do Brasil. A unidade possui três equipes de saúde da família e duas equipes de saúde bucal, juntamente com os profissionais do núcleo de apoio à saúde da família, que prestam atendimento na Atenção Primária e em urgências da Atenção Primária^(10)^.

### Participantes do estudo

A amostra foi composta por 13 cuidadoras primárias vinculadas à USF, que possuíam crianças de 0 a 6 anos. Foram excluídos cuidadores primários de crianças com necessidades especiais, devido ao comprometimento no desenvolvimento e aprendizagem já evidentes. A amostra foi selecionada intencionalmente, por conveniência, entre as cuidadoras que compareceram à unidade para consultas de puericultura, vacinação ou outros atendimentos, bem como também por meio de indicações dos agentes comunitários de saúde em visitas domiciliares. Caso não pudessem realizar a entrevista naquele mesmo dia na unidade de saúde, seria realizado o agendamento em uma organização não governamental, na mesma comunidade, no dia e horário de conveniência para os participantes. As participantes foram orientadas sobre todas as etapas do estudo, e sua anuência foi atestada pela assinatura do Termo de Consentimento Livre Esclarecido.

### Coleta de dados

Os dados foram obtidos por meio de entrevistas individuais realizadas no período de dezembro de 2022 a abril de 2023. Foram realizadas nove visitas à USF para a coleta de dados, além de três visitas prévias, realizadas para aproximação com a equipe de saúde, com a população e com o contexto do estudo. As entrevistas semiestruturadas foram conduzidas pela pesquisadora principal (mestranda) e uma auxiliar de pesquisa (aluna de iniciação científica), previamente treinadas com a técnica de realização do estudo e com os pressupostos da pesquisa qualitativa. As entrevistas foram realizadas individualmente e gravadas sem a presença de não participantes, e duraram cerca de 20 a 30 minutos. A técnica utilizada foi a entrevista semiestruturada, com o auxílio de um roteiro com perguntas abertas e um diário de campo para registro de impressões da pesquisadora logo após o término de cada entrevista.

### Análise de dados

As entrevistas foram transcritas, devolvidas às participantes por meio do aplicativo *WhatsApp^®^* e analisadas pela técnica da análise temática, mediante as seguintes etapas: transcrição; codificação dos dados iniciais; formação de subtemas; formação de temas; e categorização^(11)^.

A codificação inicial foi realizada em um arquivo do *Google Docs* ^®^ para a codificação linha a linha. Os códigos iniciais foram discutidos pela pesquisadora principal, auxiliar de pesquisa e orientadora para a sistematização, organização e criação dos subtemas e temas em reuniões periódicas. Na etapa de categorização, os dados foram organizados a partir da perspectiva dedutiva, nos componentes da *framework Nurturing Care*.

## RESULTADOS

### Caracterização das participantes

Participaram da pesquisa 13 cuidadoras primárias de crianças de 0 a 6 anos. Todas eram do sexo feminino, sendo 12 mães e uma avó. A idade variou entre 18 e 46 anos. Quanto à escolaridade e renda, a maioria tinha o ensino médio completo (dez participantes) e renda mensal de um a dois salários mínimos. Do total das entrevistadas, oito se declararam solteiras, e cinco, casadas. Quanto à religião, cinco se declararam católicas; duas se declararam cristãs/evangélicas; e seis declararam não ter religião. No que diz respeito à moradia, a maioria referiu morar com quatro pessoas. Quanto à renda, a maioria vive em média com um e meio salário mínimo. Duas famílias informaram que a renda familiar era menos de um salário mínimo. No quesito raça/cor, 11 participantes se declararam pretas ou pardas. Quanto ao tempo de cuidado com a criança, sete cuidadoras referiram não trabalhar e cuidar exclusivamente do filho, enquanto seis cuidadoras referiram trabalhar fora de casa e cuidar dos filhos no contraturno de trabalho. A informação sobre apoio familiar foi mencionada apenas pelas cuidadoras que trabalhavam. As cuidadoras que trabalhavam contavam com uma rede de apoio caracterizada por avós, tias, pais e madrinhas das crianças.

Ao se analisar os dados sociodemográficos das participantes, identificou-se a presença das mulheres como principais cuidadoras que vivem em um contexto de moradia, saneamento em condições precárias e renda limitada, além da constante exposição a riscos.

A faixa etária das crianças variou, sendo que quatro possuíam menos de 6 meses; quatro possuíam de 1-2 anos; e oito possuíam de 2-5 anos. Três cuidadoras tinham dois filhos menores de 6 anos.

### Caracterização dos dados qualitativos

As cinco categorias apresentadas estão baseadas nos eixos da *framework Nurturing Care* (Boa saúde; Nutrição satisfatória; Cuidados responsivos; Oportunidades de aprendizagem precoce; e Proteção e segurança), conforme descrito no Quadro 1. Os eixos da *framework Nurturing Care* foram explorados a partir dos temas emergidos das entrevistas individuais, os quais exploraram as vivências e práticas parentais das cuidadoras que influenciam o desenvolvimento infantil de crianças de 0 a 6 anos adstritas à ESF.

**Quadro 1:** Categorias com temas e subtemas a partir das vivências e práticas parentais e mães de crianças de 0 a 6 anos, Recife, Pernambuco, Brasil, 2023

Eixo do *framework Nurturing Care*	Temas	Subtemas	Depoimentos
**Boa saúde**	1. Situação de saúde	- Planejamento gestacional - Medo da maternidade e suas experiências	*“Essa gravidez foi um choque pra mim, foi um choque. Veio acompanhada com muito estresse”.* (E4) (Criança de 1 mês) *“Foi muito difícil, porque eu nunca quis ser mãe. Eu sempre achei lindo, mas o certo é que eu nunca quis esta responsabilidade de um filho, e hoje eu tenho que vivenciar tudo isso”.* (E5) (Crianças de 1 e 3 anos)
**Boa saúde**	2. Enfrentamento da pandemia	- Modificação da rotina das pessoas e das crianças - Modificação dos relacionamentos interpessoais e sociais dos cuidadores e crianças - Modificação na vida escolar da criança - Medidas protetivas e de prevenção contra o vírus - Medo de contaminação durante a pandemia	*“Ele veio na pandemia, porque tive um, perdi um, tive o M e, nessa gestação, eu achei que estava com COVID. No começo, tinha falta de ar, vomitando, e para mim eu achei que não era gravidez, porque, pra mim, é muito difícil ter uma gestação”.* (E1) (Criança de 2 anos) *“Trouxe mais estresse, porque ele ficava estressado, querendo sair todo instante”.* (E11) (Criança de 3 anos) *“Ensinava* (tarefas da escola) *de vez e uma vez perdida. A gente ensinava e pegava no pé dele”.* (E6) (Criança de 5 anos)
**Boa saúde**	3. Acompanhamento da criança no serviço de saúde	- Acesso e acompanhamento no serviço de saúde e uso da Caderneta da Criança	*“Eu acho que a parte do posto de saúde poderia melhorar mais na questão da criança, porque é muito difícil de conseguir atendimento”.* (E5) (Crianças de 1 e 3 anos)
**Boa saúde**	4. Desenvolvimento e comportamento da criança	- Conceito de desenvolvimento infantil - Papel parental na promoção do desenvolvimento infantil	*“Eu acho que desenvolvimento saudável é quando ela é feliz, quando ela tem a parte dela de brincar, tem a parte dela de educar, porque a gente, querendo ou não, tem que educar* [...]”. (E13) (Criança de 4 anos) *“Ela já tem um livrinho de uma amiga minha que chegou de viagem, mas ela não chega a ler; ela fica tentando rasgar”.* (E2) (Criança de 1 ano)
**Nutrição satisfatória**	1. Nutrição da criança	- Alimentação saudável da criança - Práticas de aleitamento materno	*“De manhã, quando acorda, toma um mingau que minha mãe faz. No período da tarde, ela come frutas, almoça, toma suco, lancha um pouquinho e depois de noite é mingau”.* (E2) (criança de 1 ano) *“Ele mama e toma a fórmula de noite. Porque só o peito* [...] *ele num fica*”. (E8) (criança de 1 mês)
**Cuidados responsivos**	1. Relação cuidador/criança	- Relações entre cuidador e criança e encorajamento da criança	*“Eles precisam muito da gente”.* (E1) (Criança de 2 anos) *“Se ele tá montando o quebra-cabeça, eu vou lá e vejo que ele terminou. Eu sempre digo: ‘Muito bem, parabéns’”.* (E13) (Criança de 4 anos)
**Cuidados responsivos**	2. Interação e cuidado da família com a criança e rede de apoio	- Compreendendo as práticas parentais no cuidado no dia a dia - Rede de apoio	*“Eu leio uma história, aí ele pede duas, aí eu digo: ‘Não, é uma história, tá bom?’, aí coloco a música de ninar, aí ele dorme”.* (E11) (Criança de 3 anos) *“Brinca com o pai dele. Quando está todo mundo com sono, a gente vai dormir”.* (E6) (criança de 5 anos) *“Ou eu brinco com ele ou ele vai pra casa da madrinha”.* (E11) (Criança de 3 anos) *“Não tenho o apoio do pai! Que ele deveria criar a responsabilidade de ter os filhos e cuidar deles pelo menos no final de semana”.* (E5) (Crianças de 1 e 3 anos)
**Cuidados responsivos**	3. Sentimentos com a parentalidade	- Sentimentos no cuidado com a criança	*“Eu acho que, para ser mãe, tem que ter paciência, tem que ter um pouco de experiência”.* (E4) (Criança de 1 mês) *“Prazeroso, mas muito trabalhoso. Dedicação total”.* (E1) (Criança de 2 anos)
**Oportunidades de aprendizagem precoce**	1. Relação de aprendizagem entre cuidadores e crianças no contexto domiciliar	- Participação dos cuidadores na promoção da aprendizagem - Atividades das crianças na promoção das oportunidades de aprendizagem e estímulo ao desenvolvimento	*“Eu acho que a gente tem que ensinar. A gente tem que ter paciência de ensinar* [...] *senão ele não vai aprender só”.* (E7) (Criança de 4 meses) *“Muitas vezes, é brincando que se aprende, então não todas, mas tem algumas brincadeiras que sim, estimulam muito”.* (E12) (Criança de 4 anos)
**Oportunidades de aprendizagem precoce**	2. Relação dos cuidadores com o contexto escolar	- Papel da escola na promoção do desenvolvimento e aprendizagem das crianças - Participação e acompanhamento dos cuidadores na vida escolar da criança e vivências e práticas de aprendizagem - Acessibilidade às instituições escolares	*“Ela é muito evoluída em relação à alimentação. É bem didática quando ela precisa comer. Ela pega sozinha, ela é de falar, ela é de dizer o que quer. Se ela está com fome* [...] *essas coisas assim, são bem mais vividas quando se trata de uma criança que é de creche e uma que não é de creche, que é o caso deles”.* (E5) (Crianças de 1 e 3 anos) *“Sempre que tem reuniões, está sempre presente, está sempre buscando com os professores, porque tem um grupo de professores que sempre relatam o que aconteceu o que deixou de acontecer. Ela está sempre buscando novidades”.* (E12) (Criança de 4 anos)
**Proteção e segurança**	1. Recursos financeiros e lazer	- Práticas de atividades e locais de lazer entre a família e a criança	*“Não, esporadicamente. Estou com tempo livre e com dinheiro, a gente vai”.* (E5) (Crianças de 1 e 3 anos) *“O problema é tempo. Que eu só tenho tempo no final de semana. Quando eu tenho tempo no final de semana, normalmente tento fazer o que? Cuidar da casa, fazer a limpeza, alimentação, tudo isso que é bem difícil”.* (E5) (Crianças de 1 e 3 anos)

### Categoria 1 - Boa saúde: entendendo práticas de saúde com as crianças

Na categoria “Boa saúde”, os dados apontaram as condições de saúde materna desde o planejamento e a aceitação da gravidez, as relações no cuidado, como os cuidados de higiene e a rotina de sono. O contexto da pandemia de COVID-19 foi marcado pelo medo de contaminação, e as medidas de enfrentamento estiveram voltadas a ações de prevenção e à influência na rotina da criança, como maior restrição ao ambiente doméstico e preferência por atividades ao ar livre. Além disso, houve mudanças no relacionamento social, comportamental e na vida escolar das crianças. O acompanhamento da criança nos serviços de saúde foi percebido a partir da necessidade de melhorias no acesso, incluindo consultas de puericultura e uso da Caderneta da Criança. As mães percebiam a importância da caderneta para o registro de dados da criança e para o conteúdo informativo, embora tenham descrito dificuldade para ler as informações disponibilizadas.

### Categoria 2 - Nutrição satisfatória: entendendo práticas da nutrição adequada para as crianças

Na categoria “Nutrição satisfatória”, foram relatadas pelas cuidadoras as práticas utilizadas no dia a dia referentes à alimentação e ao aleitamento materno infantil. Entre as crianças participantes, cinco estavam na faixa etária de 0 a 6 meses, porém apenas três cuidadoras relataram sobre a prática de aleitamento materno. Entre estas, uma ainda relata que o aleitamento materno exclusivo não era suficiente. O uso de industrializados e açúcares como fatores prejudiciais para uma alimentação saudável esteve presente nos relatos. Apenas uma cuidadora abordou a redução de alimentos ricos em açucares e industrializados.

### Categoria 3 - Cuidados responsivos: entendendo o conhecimento e práticas das cuidadoras no cuidado com a criança

Na categoria “Cuidados responsivos”, as cuidadoras mencionaram a percepção das necessidades das crianças a partir da compreensão de sua dependência na relação de apego e sensibilidade, bem como a menção a práticas parentais positivas, como encorajá-las a enfrentar desafios cognitivos, como montar um quebra-cabeça, e a leitura e a contação de histórias. A rede de apoio foi mencionada na participação de atividades da vida diária, sendo composta por familiares, parentes e vizinhos. Porém, algumas entrevistadas referiram ausência de membros, como a ausência do “pai” no suporte diário.

### Categoria 4 - Oportunidades de aprendizagem precoce: compreendendo as práticas dos cuidadores que viabilizam as oportunidades de aprendizagem

Na categoria “Oportunidades de aprendizagem”, as cuidadoras percebiam que as suas interações em brincadeiras e atividades diárias contribuíam para a aprendizagem da criança. Além disso, mencionaram a participação e importância da escola nesse processo, com menção ao engajamento familiar nas atividades da escola.

### Categoria 5 - Proteção e segurança: entendendo as condições de renda, de lazer e segurança das crianças

Na categoria “Proteção e segurança”, as cuidadoras mencionaram a influência dos recursos financeiros no tempo de lazer das famílias. Outro aspecto é a situação financeira das famílias, com recursos financeiros voltados particularmente à subsistência.

## DISCUSSÃO

Os resultados apresentam as vivências e práticas parentais das cuidadoras de crianças de 0 a 6 anos adstritas à ESF. As principais vivências narradas se basearam nas interações diárias de cuidado com a criança, incluindo o papel parental sobreposto às demais demandas da vida diária, como o cuidado com a casa, a relação com o desemprego ou com trabalhos que ocupam longas jornadas do dia. As práticas parentais se basearam nos relatos de ações que envolviam as conversas, brincadeiras e contação de histórias. O contexto tem relação direta com os desfechos de saúde. É importante frisar que as condições desfavoráveis do ambiente e renda das crianças e famílias podem estar relacionadas a desfechos negativos do desenvolvimento infantil.

As cuidadoras que, em sua maioria, eram as mães das crianças, mencionaram sentimentos de satisfação com a maternidade, como amor, felicidade, força, paciência, responsabilidade e prazer no cuidado da criança. Porém, foram também evidenciados os sentimentos de abdicação e de cansaço. Esse aspecto esteve relacionado tanto às mães que exerciam atividades laborais fora de casa quanto ao acúmulo de atividades do ambiente doméstico e falta de compartilhamento das responsabilidades domésticas com seus companheiros.

Esse aspecto é corroborado por estudo que aponta maiores índices de estresse, cansaço e fadiga nas mães que possuem atividades profissionais e na família. As mulheres são mais propensas a recorrer a práticas parentais autoritárias e a punições. Todo esse contexto pode ocasionar os sentimentos de culpa e incapacidade de desempenhar da melhor forma os múltiplos papéis^(12)^.

Os resultados do presente estudo foram dispostos em categorias que estão em consonância com os eixos estratégicos da *framework Nurturing Care* já mencionados neste estudo. O conjunto destes propicia os cuidados integrais à criança, protegendo, dessa forma, as crianças dos riscos que ameaçam o DI e dos impactos nos ciclos intergeracionais^(2)^.

No que concerne à categoria saúde destacaram-se os relatos a respeito das interações entre cuidadores e crianças e o acompanhamento do desenvolvimento infantil nos serviços de saúde, desde o acompanhamento pré-natal, o qual foi cercado por sentimentos de incerteza e medo durante a pandemia de COVID-19.

O acompanhamento do desenvolvimento infantil por meio das consultas de puericultura foi encontrado na realidade das participantes, embora tenha sido verbalizado a dificuldade do acesso e a limitação no uso da Caderneta da Criança como instrumento do letramento parental. Estudos apontam que a pandemia afetou principalmente as crianças que vivem em situação de vulnerabilidade, pois dificultou o acesso aos serviços de saúde e de educação e a diminuição da renda da família. Na educação, com as medidas de enfrentamento, as escolas foram fechadas, o que impactou negativamente o estado nutricional e a aprendizagem das crianças. Muitas vezes, a escola é quem fornece a alimentação diária e as oportunidades de aprendizagem e interação entre os pares^(12)^.

Além disso, os estudos mostram que houve um distanciamento entre a família e a escola, principalmente quando o ensino perpassa o modelo remoto. Muitas dessas crianças não tinham acesso à internet. Neste período, ainda aumentou a pobreza e diminuiu o emprego, o que levou ao excesso de pessoas no ambiente domiciliar e ao estresse, o que pode dificultar a prática de cuidados responsivos e consistentes pelos cuidadores^(13,14)^.

Outro aspecto explorado foi a compreensão das cuidadoras sobre o desenvolvimento infantil e os fatores que podem prejudicar o desenvolvimento da criança. Nessa perspectiva, destacou-se o conceito de desenvolvimento como a criança ser saudável, feliz, com bons hábitos relacionados à alimentação (aleitamento materno, consumo alimentar adequado), além de práticas responsivas por parte das cuidadoras, como afeto e rotinas. Em relação ao comportamento da criança, as cuidadoras mencionaram as percepções sobre habilidades motoras, como serem muito ativos, além de características ligadas ao desenvolvimento cognitivo, como prestarem atenção e gostarem dos livros e da escola, além de características comportamentais, como obediência aos cuidadores.

No tocante à categoria “Nutrição satisfatória”, as cuidadoras deste estudo mencionaram as principais práticas ligadas à nutrição, como o aleitamento materno e a alimentação complementar utilizadas no dia a dia. A nutrição infantil é um aspecto de relevância para o desenvolvimento infantil integral. Estudos apontam que a nutrição não satisfatória pode ter efeitos em vários aspectos da vida da criança, desde as questões de saúde até os riscos ao desenvolvimento infantil integral, ocasionando problemas comportamentais^(15)^. Déficits nutricionais podem resultar em baixo desempenho escolar, déficit cognitivos e doenças crônicas na vida adulta, que podem perpassar até a geração subsequente, podendo causar impactos nas condições de saúde e socioeconômicas dessas crianças a longo prazo^(16)^.

Na perspectiva dos cuidados responsivos, na realidade estudada, as ações responsivas foram encontradas nas contações de histórias antes de dormir e leitura de livros, em que as crianças interagiram e pediam mais histórias ou até mesmo criavam suas próprias histórias, além do hábito de conversar durante as interações no dia a dia, com o uso das brincadeiras adequadas para faixa etária, como mímicas, jogo das cores, jogo de dança e músicas.

Essas práticas parentais influenciam diretamente o desenvolvimento infantil, principalmente nos aspectos cognitivo e de aprendizagem das crianças, o que corresponde a práticas de cuidados responsivos^(1,6,17)^. Os cuidados responsivos partem do envolvimento e do auxílio dos pais ou outros cuidadores nas suas relações e interações com a criança, obtidas através das relações diárias, de forma sensível e atenciosa, criando, portanto, oportunidades de interações, aprendizagem com o meio, objetos e pessoas, tornando-se fundamental para obtenção de desenvolvimento infantil integral saudável^(18)^.

Na perspectiva do cuidado das crianças e das práticas parentais utilizadas, destacam-se as percepções de como o cuidador pode interferir ou auxiliar no desenvolvimento infantil. Porém, o acesso à informação não representa a sua compreensão e aplicabilidade ampla nos cuidados parentais, por motivos que incluem sobrecarga e estresse, limitação de fontes de apoio (incluindo o papel dos homens e de outros membros da família), determinantes sociais (como renda e escolaridade) e a própria falta de motivação para o aprendizado parental^(19)^.

A implementação de intervenções que apoiem os cuidadores para práticas parentais positivas e a formação de um ambiente seguro e saudável são essenciais na prática profissional dos profissionais de saúde, pois podem trazer inúmeros benefícios à saúde das crianças e sua família, principalmente no que concerne à redução de eventos e desfechos negativos^(20)^. Porém, na realidade brasileira, há dificuldade na implementação das orientações e intervenções relacionadas à aquisição de práticas positivas para o desenvolvimento infantil no contexto da Atenção Primária. Os profissionais encontram barreiras para a abordagem de temas sensíveis e que necessitam de habilidades práticas, como práticas de educação parental respeitosas, o que torna necessário haver o aperfeiçoamento e a educação permanente para qualificação das ações voltadas ao cuidado infantil^(5,21)^.

Entre as práticas parentais relatadas pelas cuidadoras, destacaram-se práticas parentais positivas como o uso de brincadeiras com desenhos e jogos educativos, músicas e contação de histórias na rotina diária das crianças. Um fato relevante é que apesar de não terem o conhecimento de práticas que incentivam a leitura e o aprendizado, foi percebido que algumas cuidadoras exercem as práticas de incentivo à leitura através do contato com livros. Não houve relatos de outros tipos de práticas negativas, como punição física e violência.

Estudos apontam os benefícios das intervenções voltadas aos estímulos das práticas parentais na perspectiva de promover conhecimento e habilidades nos cuidadores, com melhora no conhecimento dos pais e interações entre pais e filhos. Os resultados interferem também nos escores de desenvolvimento da criança, com melhores habilidades cognitiva, motora e de linguagem^(17,22)^.

Os serviços de saúde e a educação infantil foram influenciadores nas vivências e nas práticas parentais exercidas pelas cuidadoras, com acompanhamento e vigilância da saúde e aprendizagem. Estudos retratam que as práticas parentais são essenciais para criação de um ambiente propício para o desenvolvimento infantil, pois atuam como fatores de oportunidade e estimulação^(1,17,18)^.

Na categoria “Oportunidades de aprendizagem”, as cuidadoras percebiam o seu papel na aprendizagem das crianças, retratando ações de cuidado como ensinamentos e atividades lúdicas para a aprendizagem das crianças. Outro aspecto foi a percepção da importância da manutenção do acompanhamento escolar, embora a falta de tempo para um acompanhamento mais próximo e engajado tenha sido verbalizada pelas cuidadoras que exerciam atividade remunerada.

A prática parental de contação de histórias e contatos com livros pode facilitar a aprendizagem e promover os momentos de conexão entre cuidador-criança, o que pode proporcionar motivação e despertar cada vez mais interesse nas crianças. Estudos apontam que, entre os benefícios das atividades lúdicas, estão a aquisição de habilidades cognitivas, linguísticas e motoras, que estão diretamente relacionadas à aprendizagem emergente, à promoção de competências sociais e à promoção de autorregulação do comportamento e das emoções^(23)^.

Na perspectiva do acompanhamento escolar, é possível identificar que a escola é uma extensão do ambiente familiar como meio de aprendizagem. Por isso, o engajamento familiar e apoio nas atividades de aprendizagem são cruciais para a aprendizagem e desenvolvimento das crianças^(24)^.

Na categoria “Proteção e segurança”, mesmo com a situação de vulnerabilidade e de restrição econômica, algumas cuidadoras procuram promover os momentos de lazer em ambientes gratuitos, encontrados na própria comunidade e redondezas. As intervenções parentais, embora diversas em países de média e baixa renda, mostram resultados promissores particularmente a partir de estratégias de mudança de comportamento a partir de diários, lembretes por tela, contatos telefônicos, segmentos com encorajamento e uso de drama interativo (teatro e técnicas interativas)^(25)^.

No nosso estudo, as práticas parentais positivas e os cuidados responsivos foram encontrados a partir de relatos relacionados aos saberes construídos, através de suas experiências de maternidade e nas relações com seus membros familiares. Essas ações necessitam ser orientadas e ampliadas no dia a dia dessas famílias, a partir do apoio de profissionais de saúde e educação de forma integrada. Portanto, a qualificação dos profissionais de saúde para implementação de intervenções que promovem os cuidados responsivos pode gerar ganhos não só individuais, mas para toda a comunidade, seguindo uma perspectiva bioecológica defendida pela *framework Nurturing Care*, utilizada como referencial teórico para este estudo.

### Limitações do estudo

Entre as limitações do estudo, destaca-se o período adotado para a coleta de dados, haja vista que o convite e recrutamento das participantes tiveram dificuldades devido às consultas de puericultura na USF se destinarem a crianças de 0 a 2 anos. As demais estratégias de captação, como o convite para as cuidadoras que procuravam a unidade por outro motivo, vacinação e intercorrências no caso de crianças maiores, podem ter refletido em demandas adicionais relacionadas à parentalidade em momentos críticos de cuidado. A exclusão de cuidadores que possuíam crianças com necessidades especiais de saúde se deu pela justificativa de sobrecargas adicionais e maiores demandas na parentalidade em cuidadores que enfrentam esses desafios. Reconhecemos que esta é uma limitação do estudo, e pode ser campo de investigação em estudos sobre as vivências parentais.

### Contribuições para a área da enfermagem, saúde ou políticas públicas

O trabalho contribui para a discussão de práticas parentais positivas e para a ampliação de intervenções de apoio à parentalidade a partir das vivências e dos conhecimentos prévios das cuidadoras. Ações de apoio à parentalidade devem ser uma pauta prioritária para a promoção do desenvolvimento infantil, por meio da integração de componentes que superem os modelos biológicos e adotem abordagens participativas e intersetoriais, capazes de quebrar ciclos intergeracionais.

## CONSIDERAÇÕES FINAIS

As vivências e práticas parentais de cuidadoras primárias de crianças de 0 a 6 anos atendidas na ESF se alinharam aos eixos da *framework Nurturing Care*, mesmo na ausência de sua incorporação pela ESF no cenário brasileiro, devido ao contexto de vulnerabilidade vivido pelas famílias. Pode-se inferir que práticas parentais positivas de apoio à parentalidade requerem uma reorganização dos serviços de saúde a partir da implementação de intervenções que incentivem os cuidados responsivos e o desenvolvimento infantil integral.

A parentalidade foi descrita por meio de práticas positivas, com destaque para o uso de brincadeiras, músicas e contação de histórias na rotina diária das crianças como práticas positivas. Os cuidados responsivos foram descritos por meio da percepção e compreensão de como responder às ações das crianças de maneira prática e oportuna, por meio de interações sensíveis e responsivas. As cuidadoras mencionaram o papel dos serviços de saúde mais fortemente relacionado aos eixos “boa saúde” e “nutrição satisfatória”, como ações de saúde como vacinação, atendimento médico. Já a escola foi percebida como um cenário de apoio para o desenvolvimento e nas situações de aprendizagem.

## Data Availability

Os dados de pesquisa só estão disponíveis mediante solicitação.
